# Deep Learning Model for Prediction of Bronchopulmonary Dysplasia in Preterm Infants Using Chest Radiographs

**DOI:** 10.1007/s10278-024-01050-9

**Published:** 2024-03-18

**Authors:** Hao-Yang Chou, Yung-Chieh Lin, Sun-Yuan Hsieh, Hsin-Hung Chou, Cheng-Shih Lai, Bow Wang, Yi-Shan Tsai

**Affiliations:** 1 Department of Computer Science and Information Engineering, National Cheng Kung University, Tainan, 70101 Taiwan; 2 Department of Pediatrics, National Cheng Kung University Hospital, College of Medicine, National Cheng Kung University, Tainan, 704 Taiwan; 3 Institution of Medical Informatics, National Cheng Kung University, Tainan, 70101 Taiwan; 4 Institute of Manufacturing Information and Systems, National Cheng Kung University, Tainan, 70101 Taiwan; 5 Department of Computer Science and Information Engineering, National Chi Nan University, Nantou, 54561 Taiwan; 6 Institute of Information Science, Academia Sinica, Taipei, 115 Taiwan; 7 Research Center for Information Technology Innovation, Academia Sinica, Taipei, 115 Taiwan; 8 Department of Medical Imaging, National Cheng Kung University Hospital, Tainan, 701401 Taiwan; 9 Department of Medical Imaging, National Cheng Kung University Hospital, College of Medicine, National Cheng Kung University, Tainan, 704 Taiwan

**Keywords:** Artificial intelligence, Preterm infants, Infant chronic lung disease, Lung region segmentation, Prognosis

## Abstract

Bronchopulmonary dysplasia (BPD) is common in preterm infants and may result in pulmonary vascular disease, compromising lung function. This study aimed to employ artificial intelligence (AI) techniques to help physicians accurately diagnose BPD in preterm infants in a timely and efficient manner. This retrospective study involves two datasets: a lung region segmentation dataset comprising 1491 chest radiographs of infants, and a BPD prediction dataset comprising 1021 chest radiographs of preterm infants. Transfer learning of a pre-trained machine learning model was employed for lung region segmentation and image fusion for BPD prediction to enhance the performance of the AI model. The lung segmentation model uses transfer learning to achieve a dice score of 0.960 for preterm infants with $\le$ 168 h postnatal age. The BPD prediction model exhibited superior diagnostic performance compared to that of experts and demonstrated consistent performance for chest radiographs obtained at $\le$ 24 h postnatal age, and those obtained at 25 to 168 h postnatal age. This study is the first to use deep learning on preterm chest radiographs for lung segmentation to develop a BPD prediction model with an early detection time of less than 24 h. Additionally, this study compared the model’s performance according to both NICHD and Jensen criteria for BPD. Results demonstrate that the AI model surpasses the diagnostic accuracy of experts in predicting lung development in preterm infants.

## Introduction

Currently, the development of deep learning technology in medical imaging is maturing rapidly, and many tasks related to AI in medical imaging have been studied [1–5]. Applying deep learning techniques to medical and healthcare services improves the quality of medical services, and diseases can be diagnosed more accurately and quickly. Bronchopulmonary dysplasia (BPD) occurs frequently in preterm infants, and impaired lung development can lead to pulmonary vascular disease, affecting the integrity of lung function [6]. In the past, doctors diagnosed BPD through chest radiographs by their subjective assessment. The different clinical experiences and physical conditions of clinicians may lead to different subjective assessments of BPD. A deep learning-based assistance system for effective, efficient prediction of lung development in preterm infants may help clinicians make more accurate diagnoses and improve medical care quality.

In recent years, deep learning techniques have achieved remarkable results in many fields and are still expanding [7]. Among them, Convolutional Neural Networks (CNN) is currently the most popular deep neural network. It has demonstrated outstanding performance in image recognition and performs better than humans in completing many tasks [5, 8–10]. Despite the use of AI-based lung segmentation on adult lungs by many researchers, few studies have tackled the challenge of segmenting lungs in preterm infants and children due to difficulties in data collection, smaller lung areas, whiter lung attenuation, and many neonatal lines [11, 12]. Previous studies have investigated the use of late chest radiographs in predicting BPD in premature infants, specifically at the postnatal age of 7 days or 28 days. These studies primarily focused on neonatal chronic lung disease and respiratory disorders [13–15]. The present study aims to use deep learning techniques to segment the lung areas and forecast lung development outcomes at an early stage, specifically within less than 24 h of gestational age, on preterm chest radiographs. By applying deep learning technology to segment lungs and developing a BPD prediction model that targets lung parenchyma, our study makes a valuable contribution to early detection and prediction for infants at risk of BPD.

**Fig. 1:**
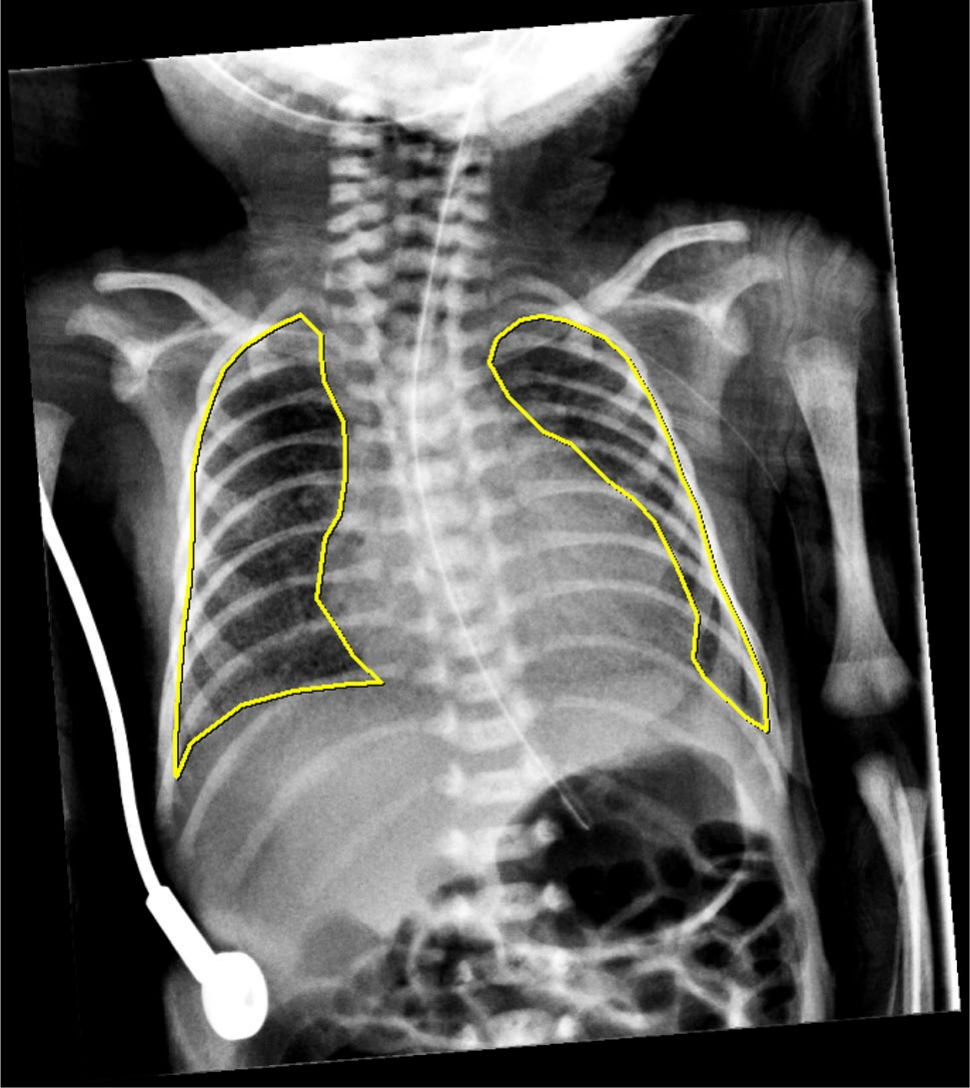
An example of the labeling for lung areas

**Fig. 2:**
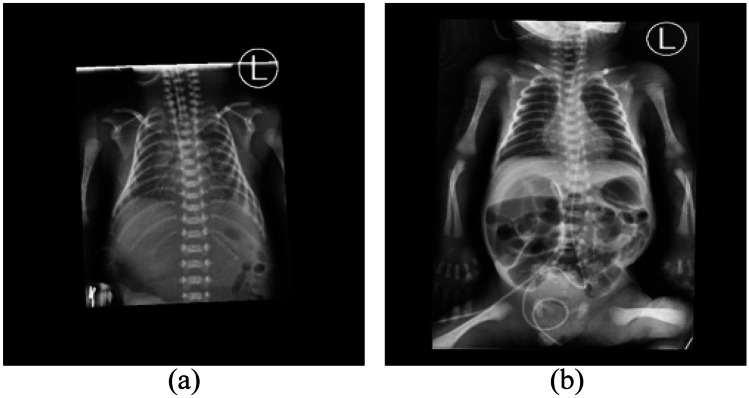
**a.** The chest radiograph taken at 0 h postnatal age in a preterm infant and treated with surfactant immediately **b.** The chest radiograph of the same infant taken at 47 h postnatal age after treatment with surfactant showed improving lung aeration

## Materials and Methods

### Study Design, Sample, and Ethics Statement

This study is a retrospective analysis of the data of newborn infants at National Cheng Kung University Hospital from January 2015 to December 2020. This included one cohort of preterm infants born before 37 weeks of pregnancy, consisting of 2124 radiographs from 154 male and 138 female infants. The other cohort comprised 88 term infants delivered between 37 and 42 weeks. Serial chest radiographs of all preterm infants were collected during hospitalization, and those of term infants were taken on the first day at birth due to transient tachypnea of newborns or other reasons that required a survey for lung conditions.

The study protocol was approved by the Institutional Review Board of National Keng Chung Hospital (IRB, B-ER-110-366). The IRB also approved a waiver of informed consent because of the anonymity of retrospective data of the included infants.

#### Lung Segmentation Dataset

The lung segmentation dataset comprises 1491 images, including 1403 chest radiographs from the preterm infant cohort that were easy to label. Another 88 chest radiographs from the term infant cohort were also added to supplement the dataset. A radiology technician with 30 years of experience (CSL) labeled the lung fields of chest radiographs along the inner chest wall and the mediastinal border. The labeling was re-checked and corrected by a pediatric radiologist (YST) with 20 years of experience. An example of the labeling for lung areas is shown in Fig. 1. The lung field was labeled along the inner chest wall and the mediastinal border.

#### BPD Prediction Dataset

Pulmonary surfactant is comprised of a complex mixture of phospholipids and proteins secreted by type II pneumocytes [16]. These surfactant granules can typically be identified as early as 20 weeks of gestation and are present in adequate amounts until the 34th week. However, when there is a deficiency of surfactant related to prematurity, it can result in hyaline membrane disease. Such a deficiency can have several impacts, including an increase in alveolar surface tension, which causes resistance to inflation and leads to the collapse of the alveoli during expiration. In current practice, neonates at risk for hyaline membrane disease are treated with respiratory support and surfactant replacement therapy. However, it is essential to note that respiratory support itself can cause damage to the alveoli due to the shear stresses that are exerted on the alveolar walls. This can eventually lead to bronchopulmonary dysplasia (BPD), a chronic lung disease that is prevalent in preterm infants and can result in alveolar simplification [17]. As a result, it is crucial to initiate continuous positive airway pressure (CPAP) from birth for infants at risk of developing RDSs [18].

A total of 1021 images taken at $\le$ 168 h postnatal age were collected as the BPD prediction dataset to build an early image biomarker for a BPD predictive AI model. Another 1103 chest radiographs were excluded because the images were taken >168 h postnatal period and images were superimposed by neonatal lines, pneumothorax, or massive pleural effusion. As already known, the therapeutic use of surfactants improves the clinical course and outcomes are prematurity in about 73% of cases [19]. The therapeutic effect of surfactant was shown in one preterm infant with improved lung aeration, as seen in Fig. 2. The time of surfactant application is usually 1 day after birth. The BPD prediction dataset was further divided into two subgroups of $\le$ 24 h postnatal with 505 images and 25–168 h postnatal with 516 images after delivery to analyze the performance of the BPD prediction AI model in these two different periods in order to alleviate the treatment effect from surfactant. The prognosis of lung development was evaluated using two criteria, one from the National Institute of Child Health and Human Development (NICHD) and the other from the Jensen Grade system [20, 21]. The two criteria for grading BPD severity were summarized and compared as shown in Table 1. The cohort was assessed at 36 weeks of postmenstrual age or upon discharge home according to the status of $O_{2}$ consumption.

**Table 1:** Definition of Bronchopulmonary Dysplasia — Diagnostic Criteria

	NICHD		Jensen Grade	
Gestational Age	< 32wk	$\ge$ 32wk		No target group
Time point of assessment	36wk PMA or discharge to home, whichever comes first	> 28d but < 56d postnatal age or discharge to home, whichever comes first		36wk PMA or discharge to home, whichever comes first
No BPD	No treatment with FiO2 > 21% for $\ge$ 28d			Breathing room air
With BPD	Treatment with FiO2 > 21% for at least $\ge$ 28d			Regardless of the prior duration or current level of oxygen therapy
Mild	Breathing room air		Grade 1	NC $\le$ 2L/min “low flow”, Any FiO2
Moderate	FiO2 < 30%		Grade 2	NC > 2L/min “high flow”, Any FiO2 nCPAP or NIPPV, Any FiO2
Severe	FiO2 $\ge$ 30% and/or positive pressure (PPV or nCPAP)		Grade 3	Invasive PPV, Any FiO2

*NICHD* The National Institute of Child Health and Human Development, *PMA* postmenstrual age, *BPD* bronchopulmonary dysplasia, *FiO2* inspiratory fraction of oxygen, *NC* nasal cannula, *NIPPV* noninvasive positive pressure ventilation, *PPV* positive pressure ventilation, *nCPAP* nasal continuous positive airway pressure

### The AI Flowchart of Lung Segmentation and BPD Prediction

**Fig. 3:**
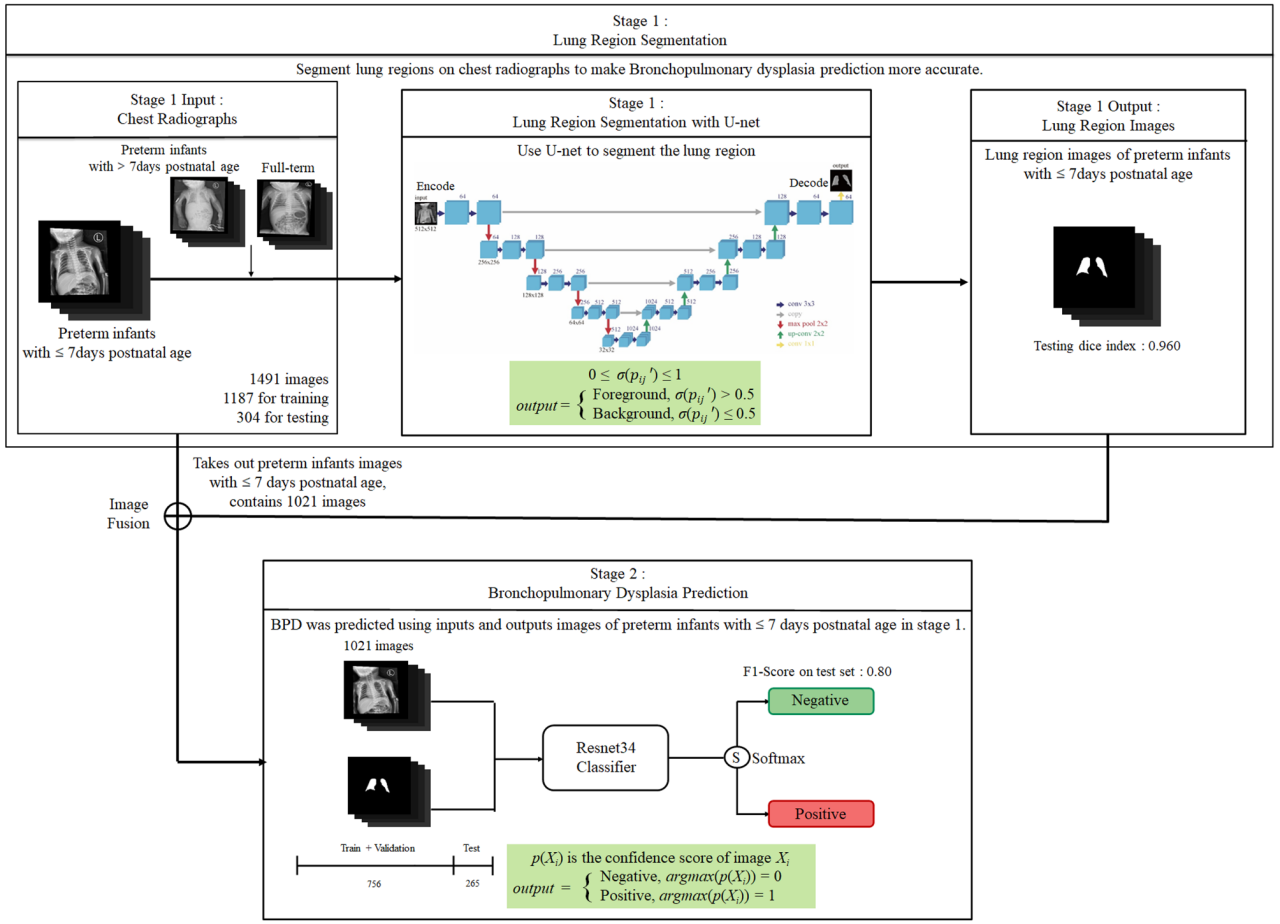
The flowchart of the AI model for lung segmentation and BPD prediction

Deep learning technologies were used to model lung segmentation in chest radiographs and BPD prediction in preterm infants. Since the development of the lungs is an essential indicator of BPD, the design concept of our model is to segment the lungs and let the model training pay more attention to the lung features. The flowchart of the method is shown in Fig. 3. In the first stage, the lung area was segmented from the chest radiographs and then, in the second stage, the original radiographs were used and the images of segmented lung areas of preterm infants at < 168 h of postnatal age for BPD prediction.

#### Lung Segmentation AI Modeling

**Fig. 4:**
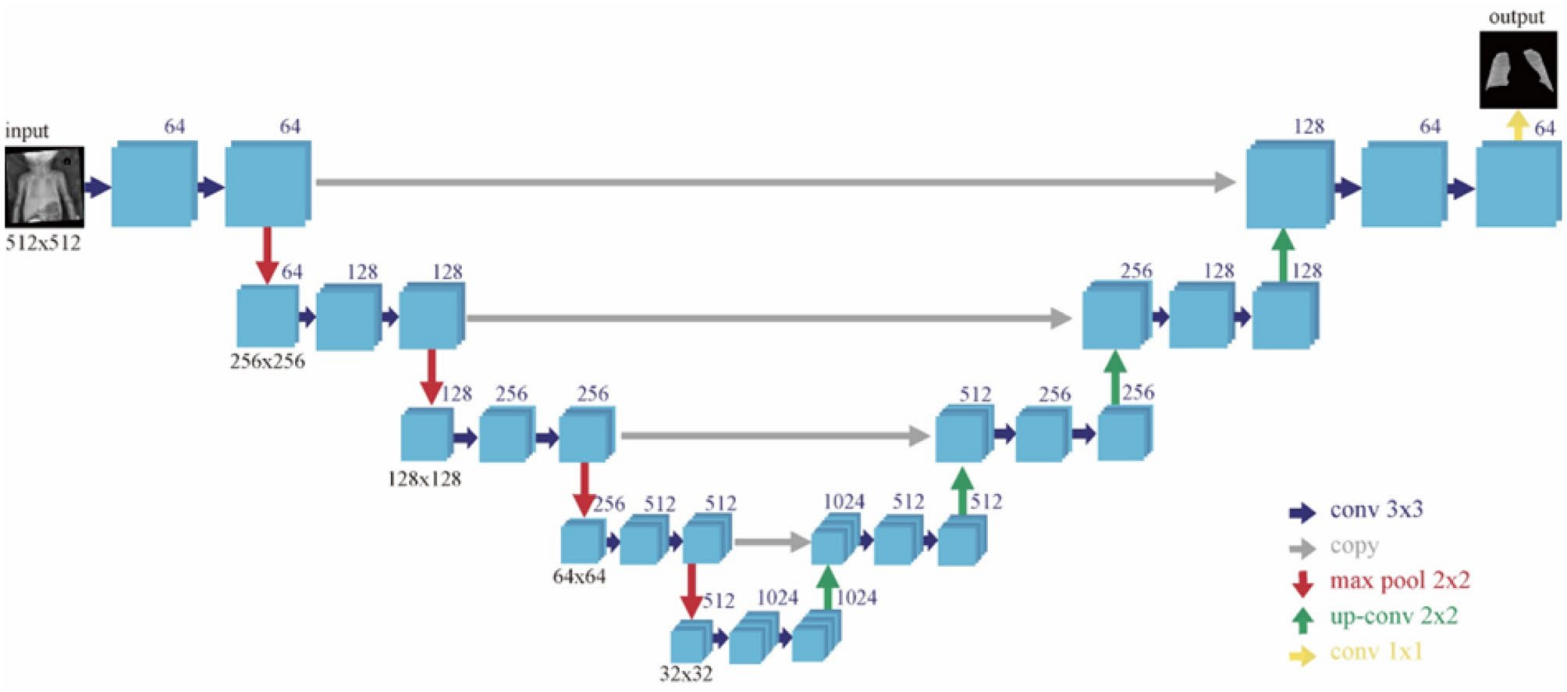
The structure of U-Net model

The U-Net model is known to perform well in semantic segmentation, and was chosen in the present study for lung area segmentation [22, 23]. U-Net mainly consists of convolution layers, down-sampling layers (pooling layer), and up-sampling layers, forming a shape like a “U”, hence its name. The structure of the U-Net is shown in Fig. 4. Additionally, to overcome the smaller data size in the cohort, transfer learning was used, which captures image features from one of Kaggle’s public adult lung segmentation datasets (https://www.kaggle.com/datasets/newra008/lung-mask-image-dataset). The dataset from Kaggle provides image features for the thoracic cage border, allowing for the separation of lung parenchyma, mediastinum, and soft tissue in the chest wall. This anatomy in chest x-ray images shows minimal differences between neonates and adults. Therefore, we initially apply the training weight of the U-Net model from the adult dataset and then fine-tune it with the neonatal dataset.

#### BPD Prediction AI Modeling

Results of the lung segmentation model and parameter tuning were used to enhance the performance of BPD prediction. To address the issue of imbalanced datasets, the weighted random sampler was used. The formula for calculating class weights was as follows:

1 $$\begin{aligned} Weight_{i} = \frac{N}{C \times N_{i}}, \end{aligned}$$

where *N* represents the total number of samples in the dataset, *C* represents the number of classes in the dataset, and $N_{i}$ represents the number of samples in class *i*.

To facilitate focused learning of image features from lung regions, we employed feature fusion techniques. Specifically, the original radiographs were concatenated with lung regions segmented by U-Net model and this concatenated image was used as input for training ResNet classifier. For each image, in the feature fusion stage, the following operation was performed:

2 $$\begin{aligned} input = [X_{i}, U(X_{i})], \end{aligned}$$

where $X_{i}$ represents a radiograph and $U(X_{i})$ is the lung area obtained by sending the radiograph into the U-Net model for segmentation. $[X_{i}, U(X_{i})]$ represents the concatenation of the two images.

**Fig. 5:**
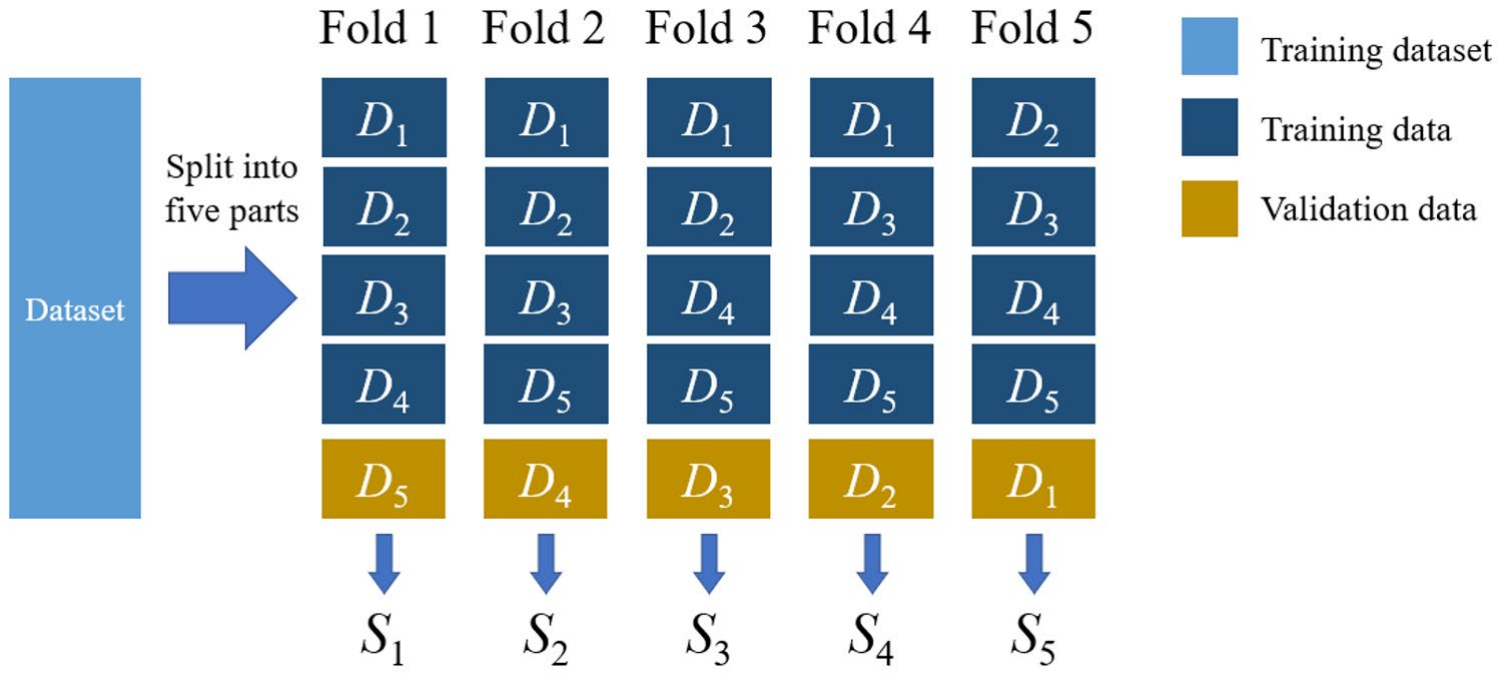
Fivefold cross-validation method

Building the model required approximately 200 epochs from the start of training until it was fully converged. We reduced the learning rate by multiplying it by 0.3 every 40 epochs to help the model escape from the saddle point and find a better solution, improving its performance. The five-fold cross-validation method was used as shown in Fig. 5.

**Fig. 6:**
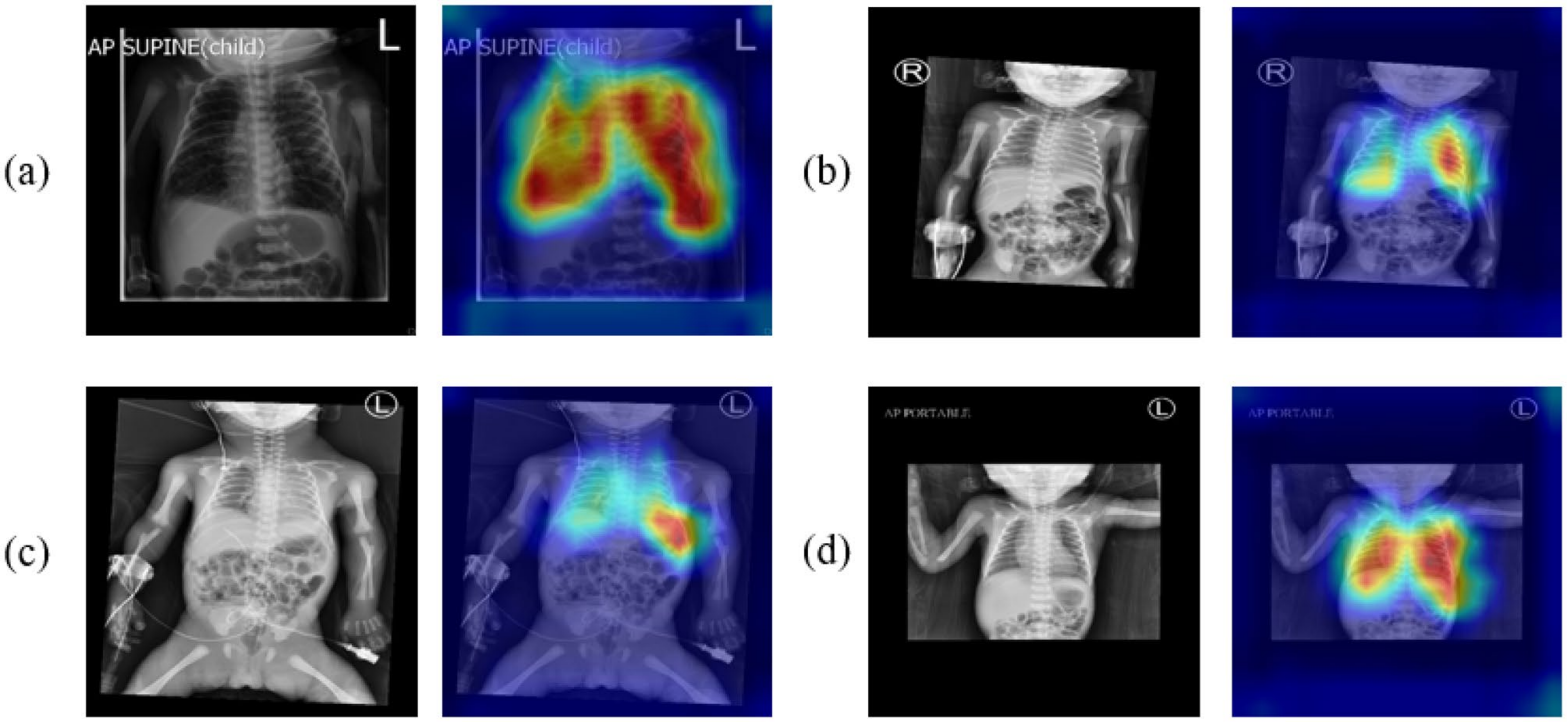
The examples of preterm chest radiographs show the heat maps with good attention to lung areas. **a**–**b** Chest radiographs for cases with BPD. **c**–**d** Chest radiographs for cases without BPD

We divided the training set into five equal parts and chose one as the validation set, while the others were used for training. The model was trained with the above data distribution to get five results. The average of the results was considered the final performance of our model. We used the heatmap on the BPD prediction model to provide interpretable visualization for the lung areas to determine whether the lung areas contained the critical features or not. Some examples of preterm chest radiographs with heatmaps are shown in Fig. 6.

### Performance Evaluation

Here, in this subsection, we introduce the evaluation criteria and formulas used for the lung segmentation model and the BPD prediction model. First of all, we introduce the notations *TP*, *FP*, *TN*, and *FN*.

- True positives (TP): The prediction was yes, and the true value is yes.
- True negatives (TN): The prediction was no, and the true value is no.
- False positives (FP): The prediction was yes, but the true value was no.
- False negatives (FN): The prediction was no, but the true value is yes.

#### Lung Segmentation Evaluation Criteria

We used images from three subgroups: term infants, prematurity $\le$ 168 h postnatal age, and prematurity > 168 h postnatal age to test the segmentation model. The performance of the model was evaluated using the dice index, which is commonly used in image segmentation tasks. The formula is as follows:

3 $$\begin{aligned} dice=\frac{2 \times TP}{2 \times TP+FP+FN} ~, \end{aligned}$$

where *TP*, *FP*, *TN*, and *FN* are calculated for all pixels of an image. The dice index ranges from 0 to 1, with higher values indicating better performance of the model.

#### BPD Prediction Evaluation Criteria

The target subgroup in the BPD prediction dataset consists of images taken $\le$ 168 h postnatal age. We have selected five common evaluation indicators to assess the performance of the BPD prediction model, which are sensitivity, specificity, precision, accuracy, and F1-score. The formulas for these indicators are as follows:

4 $$\begin{aligned} Sensitivity = \frac{TP}{ (TP + FN)} ~, \end{aligned}$$

5 $$\begin{aligned} Specificity = \frac{TN}{ (FP + TN)} ~, \end{aligned}$$

6 $$\begin{aligned} Precision = \frac{TP}{(TP + FP)} ~, \end{aligned}$$

7 $$\begin{aligned} Accuracy = \frac{(TP + TN)}{ (TP + FP + TN + FN)} ~, \end{aligned}$$

8 $$\begin{aligned} F1 \text{- }Score = \frac{2 \times Sensitivity \times Precision}{ Sensitivity+Precision} ~. \end{aligned}$$

where *TP*, *FP*, *TN*, and *FN* are calculated for all classifications of the test data.

#### Human-Machine Competition

We invited doctors with extensive experience and professional knowledge to perform lung prognosis predictions simultaneously with our model. We compared the performance of the BPD prediction model with that of experts in a human-machine competition dataset. One expert was a neonatologist (YCL) with 20 years of experience, and the other was a radiologist (YST) with 20 years of experience. The neonatologist used the direct visual assessment of the chest radiographs to determine whether BPD was present. The radiologist used four visual gradings to assess the severity of interstitial lung disease and predict BPD [24]. The visual grading for the severity of BPD was as follows: Grade 1 — No demonstrable abnormality, Grade 2 — Granular infiltration, Grade 3 — Diffuse streaky interstitial thickening, and Grade 4 — Diffuse coarse interstitial thickening. Method one defined grade 3 and 4 interstitial changes as interstitial pneumonia patterns for BPD; however, method two determined only grade 4 interstitial changes to indicate BPD.

## Results

### Experimental Environment and Parameters

All experiments were conducted on an NVIDIA Tesla V100 GPU with 32 GB of memory. As the original images were of different sizes, we preprocessed them by resizing all of them to 512 x 512 before training the model. For the lung segmentation model, we used the Root Mean Squared Propagation (RMSProp) optimizer, a batch size of 4, a learning rate of 0.001, and trained the model for 200 epochs. For the BPD prediction model, we used Stochastic Gradient Descent (SGD) as the optimizer, a batch size of 32, an initial learning rate of 0.0008 that gradually decreased, and trained the model for 200 epochs. The preterm infant cohort comprised 154 males and 138 females, with a mean gestational age of 26 ± 2.379 weeks (range: 22–30 weeks) and a mean birth weight of 840 ± 279 g (range: 420–1780 g). A total of 1021 preterm chest radiograph images taken $\le$ 168 h postnatal age were included in the BPD prediction dataset and were labeled according to the NICHD or Jensen grade system. The number of chest radiograph images in the BPD prediction dataset is shown in Table 2, with 579 images labeled as BPD by the NICHD criteria and only 457 labeled by the Jensen grade criteria.

**Table 2:** The number of images in the BPD prediction dataset labeled as whether with BPD or not

NICHD				
$\le$ 24 h	Train	Validation	Test	Total
BPD ($+$)	178	45	79	302
BPD (−)	108	27	68	203
25–168 h				
BPD ($+$)	176	44	57	277
BPD (−)	142	36	61	239

Jensen Grade				
$\le$ 24 h	Train	Validation	Test	Total
BPD ($+$)	152	38	53	243
BPD (−)	135	33	94	262
25–168 h				
BPD ($+$)	133	33	48	214
BPD (−)	186	46	70	302

*BPD* bronchopulmonary dysplasia, *NICHD* The National Institute of Child Health and Human Development

### Segmentation Performance

**Fig. 7:**
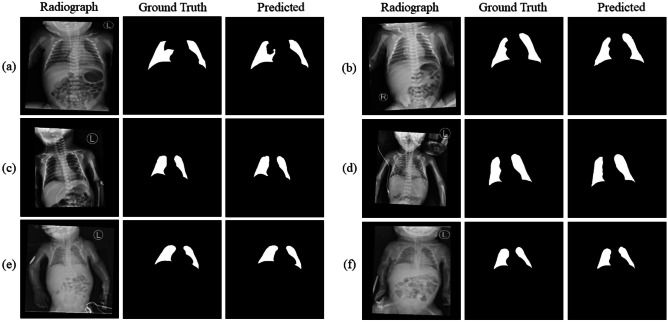
The figure shows the segmentation result including original image, ground truth masks, and predicted masks. **a**–**b** Full-term infants. **c**–**d** Preterm infants with $\le$ 168 h postnatal age. **e**–**f** Preterm infants with > 168 h postnatal

We split the segmentation dataset into training and testing datasets at a 4:1 ratio. The U-Net model for segmentation achieves a Dice index of 0.902, 0.931, and 0.920 in the subgroups of full-term, $\le$ 168 h postnatal age, and > 168 h postnatal age, respectively. The applied transfer learning method outperforms the original U-Net with a Dice index of 0.922, 0.960, and 0.950 in these three individual subgroups. The segmented lung areas on the chest radiographs of preterm infants with $\le$ 168 h and > 168 h postnatal age were illustrated, as shown in Fig. 7. The preterm chest radiographs taken $\le$ 168 h postnatal age achieve the best performance, as shown in Table 3.

**Table 3:** The segmentation performance from testing data in U-Net, transfer learning and different postnatal age

	Dice Index		
Method	Full-term	$\le$168 h	> 168 h
U-Net	0.902	0.931*	0.920
Transfer learning	0.922	0.960*	0.950

*Bold entries refer to maximum values

### BPD Prediction Performance

We conducted fivefold cross-validation on the test set of 0–24 h and 25–168 h for the evaluation of NICHD and Jensen Grade criteria, and the average F1-score results were approximately 0.8, as presented in Table 4.

**Table 4:** The fivefold validation result of BPD prediction testing dataset

	Fold 1	Fold 2	Fold 3	Fold 4	Fold 5	Average
NICHD ($\le$ 24 h)						
Sensitivity	0.886	0.899	0.886	0.899	0.886	0.891
Specificity	0.765	0.779	0.750	0.765	0.765	0.765
Precision	0.814	0.826	0.805	0.816	0.814	0.815
Accuracy	0.830	0.844	0.823	0.837	0.830	0.833
F1-score	0.806	0.822	0.797	0.813	0.806	0.809
NICHD (25–168 h)						
Sensitivity	0.825	0.842	0.842	0.807	0.860	0.835
Specificity	0.770	0.787	0.770	0.803	0.754	0.777
Precision	0.770	0.787	0.774	0.793	0.766	0.778
Accuracy	0.797	0.814	0.805	0.805	0.805	0.805
F1-score	0.797	0.814	0.807	0.800	0.810	0.805
Jensen Grade ($\le$ 24 h)						
Sensitivity	0.766	0.777	0.755	0.777	0.745	0.764
Specificity	0.736	0.755	0.736	0.717	0.774	0.744
Precision	0.837	0.849	0.835	0.830	0.854	0.841
Accuracy	0.755	0.769	0.748	0.755	0.755	0.757
F1-score	0.800	0.811	0.793	0.802	0.796	0.800
Jensen Grade (25–168 h)						
Sensitivity	0.771	0.771	0.757	0.786	0.757	0.769
Specificity	0.813	0.792	0.813	0.792	0.792	0.800
Precision	0.857	0.844	0.855	0.846	0.841	0.849
Accuracy	0.788	0.780	0.780	0.788	0.771	0.781
F1-score	0.812	0.806	0.803	0.815	0.797	0.807

*BPD* bronchopulmonary dysplasia, *NICHD* The National Institute of Child Health and Human Development

### Human-Machine Competition Result

The results of the human-machine competition are shown in Table 5. The AI model achieved better diagnostic performance than these two experts, with an accuracy of 0.75 to 0.83, and maintained a stable performance in chest radiographs taken at $\le$ 24 h postnatal age and from 25 to 168 h postnatal age. The two experts showed poor diagnostic performance in images taken $\le$ 24 h postnatal age compared to images taken from 25 to 168 h postnatal age due to obscure lung aeration before the administration of surfactant. The BPD prediction AI model can predict lung development well and performs better than experienced doctors with professional knowledge, especially on the dataset with $\le$ 24 h postnatal age.

**Table 5:** Human-machine competition result for BPD prediction AI model

	NICHD				Jensen Grade			
	Expert-A	Expert-B-M1^b^	Expert-B-M2^a^	Model	Expert-A	Expert-B-M1^b^	Expert-B-M2^a^	Model
$\le$24 h								
Sensitivity	0.588	0.485	0.706	0.765*	0.543	0.383	0.681	0.764*
Specificity	0.595	0.810	0.481	0.891*	0.604	0.774*	0.528	0.743
Precision	0.556	0.688	0.539	0.858*	0.708	0.750	0.719	0.841*
Accuracy	0.592	0.660	0.585	0.833*	0.565	0.524	0.626	0.757*
F1-Socre	0.571	0.569	0.612	0.809*	0.615	0.507	0.700	0.800*
25–168 h								
Sensitivity	0.639	0.541	0.869*	0.777	0.586	0.457	0.771*	0.769
Specificity	0.702	0.860*	0.561	0.835	0.688	0.813*	0.500	0.800
Precision	0.696	0.805	0.680	0.835*	0.732	0.781	0.692	0.849*
Accuracy	0.670	0.695	0.720	0.805*	0.627	0.602	0.661	0.781*
F1-Socre	0.667	0.647	0.763	0.805*	0.651	0.577	0.730	0.807*

*BPD* bronchopulmonary dysplasia, *NICHD* The National Institute of Child Health and Human Development

*Bold entries refer to maximum values

^a^Expert-B-M2 defined grade 4 interstitial changes as interstitial pneumonia patterns for BPD

^b^Expert-B-M1 defined grade 3 and 4 interstitial changes as interstitial pneumonia patterns for BPD

## Discussion

Approximately 15 million infants are born prematurely annually, that is, before completing 37 weeks of gestation. Prematurity is the leading cause of death in children under 5 years old. Bronchopulmonary dysplasia is a common chronic lung complication in premature infants. BPD diagnosis in prematurity is based on two criteria: the NICHD evaluation and the Jensen Grade evaluation criteria, which depend on the oxygen consumption status at 36 weeks postmenstrual age or discharge to home. The Jensen grade achieved an accuracy of 0.79 and is predictive of late death or severe respiratory morbidity [20]. Clinicians in neonatal intensive care units (NICUs) use routine chest radiographs to evaluate prematurity and to check chest conditions or intravascular catheter location. Several scoring methods are used for assessing BPD by chest radiographs [24–26]. The latest method used visual gradings of the chest radiograph on day 7 to evaluate prematurity interstitial lungs to predict BPD and achieved sensitivity and specificity of 25% and 98%, respectively [24]. In contrast, our expert-B achieved 48% and 81% at $\le$ 24 h postnatal age and 54% and 86% from 25 to 168 h postnatal age, respectively, using the same visual scoring method. However, this subjective visual grading method for BPD evaluation in prematurity reveals problems with a high false negative rate and an inconsistent result.

Surfactants have been shown to improve the clinical course and outcome of prematurity in approximately 73% of cases, with uniform or asymmetric improvement of pulmonary aeration leading to good long-term outcomes. However, chest radiographs taken $\le$ 24 h postnatal age without surfactant or with only early pulmonary aeration effect just after therapeutic surfactant may obscure the accuracy in human interpretation, as shown in our data for the two experts. Nevertheless, the newly developed BPD prediction model achieves a good balance between 76% and 78% in sensitivity and between 84% and 89% in specificity in both follow-up periods.

Artificial intelligence technologies have been applied to medical images of chest radiographs, resulting in better diagnostic accuracy, faster reading times, and improved overall diagnostic performance for general physicians [27]. Lung segmentation has been shown to perform well, with a dice coefficient of 0.989 for adult chest radiographs and a dice coefficient of 0.948 for toddler chest radiographs [11, 12]. Our lung segmentation model achieves dice coefficients of 0.922, 0.960, and 0.950 in subgroups of full-term infants, infants with $\le$ 168 h postnatal age, and infants with > 168 h postnatal age, respectively. However, the dice coefficient for full-term infants is only 0.922 due to the lack of images in the dataset. Consequently, the model may not have learned enough features to segment more accurately. Notably, lung segmentation can significantly improve the diagnosis of diseased lungs, such as bacterial or viral pneumonia [12]. Preterm infants are at risk of lung injury due to inflammation, disrupted and impaired alveoli, fibrotic lung, angiogenesis dysfunction, and eventual progression to BPD. Our study applies deep learning to segment lungs using preterm chest radiographs and builds a BPD prediction model focusing on lung parenchyma. The attention heatmap also illustrated the lung areas that contributed important features for AI modeling. The experience of lung segmentation in preterm infants is limited mainly due to the challenges in data collection. As there is a lack of lung images in preterm infants, we were unable to find an external validation set to test our model. Instead, we invited experienced physicians to participate in a human-machine competition and compared their results with those of our BPD prediction AI model using the same test set. Our BPD prediction model performed well on both the images with $\le$ 24 h postnatal age and those with > 24 h postnatal age, outperforming experienced doctors with professional knowledge, especially on the $\le$ 24 h postnatal age dataset.

The current treatment strategy for BPD mainly relies on supportive care with oxygen supply and pharmacotherapy. Recently, stem cell therapy for BPD in newborns has shown promise in reducing complications [28], especially when administered early on postnatal day 3, with better outcomes than at a later period of day 10 [29]. Visual grading from the chest radiograph as early as day 7 can predict BPD or death before 36 weeks of postmenstrual age [24]. However, we need to develop a more accurate, personalized, and early image biomarker, ideally within < 24 h, to predict BPD, allowing us to plan stem cell therapy as soon as possible.

The present study has several limitations. First, the retrospective design limits the extent to which results can be generalized to other populations. Also, the study cohort only includes medical images of neonatal chest radiographs from one medical center with limited data, which also limits the generalization of results. Due to the lack of lung images in preterm infants, we could not find an open data source for an external validation set to test our model. Instead, we invited experienced physicians to compare the results with those of our model using the same test set. Experience in lung segmentation in preterm infants and children is limited due to the difficulties in data collection, variable thymus shadow, smaller lung areas, whiter lung attenuation, and many neonatal lines. As we use the NICHD and Jensen grade criteria adjusted by the O2 demand situation at 36 weeks of postmenstrual age, or while discharged home, the target gold standard depends on the quality of newborn care. Variations in care quality may affect the performance of the BPD prediction model. Future studies should involve more medical centers with specialists in neonatology and services with NICUs to accumulate more data and test the performance of the BPD prediction model. Retraining local image datasets and fine-tuning parameters to fit the care quality in individual sites is crucial for integrating this early image biomarker into daily routine care. Federated learning, which trains an algorithm through multiple decentralized edge devices without exchanging data, is another possible alternative solution.

## Conclusion

The present study aims to use deep learning to segment lungs based on preterm chest radiographs to develop a BPD prediction model with an 80% accuracy rate. Specifically, AI technologies were applied to medical images of chest radiographs to improve the diagnostic accuracy of BPD in preterm infants. Deep learning was used for lung segmentation of preterm chest radiographs to build a BPD prediction model by focusing on lung parenchyma. Study results showed that the AI model performed better than experienced doctors in predicting lung development in preterm infants, and illustrates the potential of AI in improving the diagnosis of BPD in preterm infants.

## Data Availability

The datasets used and analyzed during the current study are available from the corresponding author upon reasonable request.
